# Dynamics of Tropomyosin in Muscle Fibers as Monitored by Saturation Transfer EPR of Bi-Functional Probe

**DOI:** 10.1371/journal.pone.0021277

**Published:** 2011-06-20

**Authors:** Roni F. Rayes, Tamás Kálai, Kálmán Hideg, Michael A. Geeves, Piotr G. Fajer

**Affiliations:** 1 Institute of Molecular Biophysics, Florida State University, Tallahassee, Florida, United States of America; 2 National High Magnetic Field Laboratory, Tallahassee, Florida, United States of America; 3 Department of Organic and Medicinal Chemistry, University of Pécs, Pécs, Hungary; 4 School of Biosciences, University of Kent, Canterbury, United Kingdom; National Institute for Medical Research, Medical Research Council, London, United Kingdom

## Abstract

The dynamics of four regions of tropomyosin was assessed using saturation transfer electron paramagnetic resonance in the muscle fiber. In order to fully immobilize the spin probe on the surface of tropomyosin, a bi-functional spin label was attached to i,i+4 positions via cysteine mutagenesis. The dynamics of bi-functionally labeled tropomyosin mutants decreased by three orders of magnitude when reconstituted into “ghost muscle fibers”. The rates of motion varied along the length of tropomyosin with the C-terminus position 268/272 being one order of magnitude slower then N-terminal domain or the center of the molecule. Introduction of troponin decreases the dynamics of all four sites in the muscle fiber, but there was no significant effect upon addition of calcium or myosin subfragment-1.

## Introduction

The activation of muscle contraction is a process that involves multiple proteins. In skeletal muscle it is the troponin (Tn) complex and tropomyosin (Tm) that regulate contraction in a calcium (Ca^2+^) dependent manner. The generally accepted “steric-block” model [1], [2], [3] postulates a Ca^2+^-induced conformational change in troponin C (TnC), propagating to troponin I (TnI) and troponin T (TnT), which leads to the movement of Tm that exposes the myosin binding sites on actin and allows myosin heads to attach and produce force. Biochemical studies [4] of Geeves and collaborators established presence of *blocked*, *closed* and *open* states which might correspond to the structural states visualized by electron microscopy [5], [6], [7], [8] characterized by different positions of Tm on the thin filament. To capture transitions between these conformations was more difficult; Förster Resonance Energy Transfer (FRET) [9], [10], [11], [12], [13] did not find evidence for the movement of Tm relative to actin and raised the possibility of the intrinsic flexibility of tropomyosin masking the “rigid body” movements of Tm on actin. A model involving the dynamics of Tm has been proposed, in which variations in the amplitude of tropomyosin vibrations drive the change in its radial position on actin [14]. There is relatively little information about Tm dynamics in muscle fiber. An early attempt measured proximity between the two strands of Tm using pyrene excimer fluorescence and observed no effect of Tn nor of Ca^2+^ [15]. More direct measurement of Tm dynamics using spin labels [16] where nanosecond mobility in the reconstituted fibers was shown to be modulated by addition of myosin subfragment-1 (S1) with no effect of Ca^2+^. Similar results were obtained by phosphorescence anisotropy [17].

The present study extends these latter efforts in two significant ways. First, bi-functional labels that are immobilized with dual attachment to i,i+4 cysteine sites engineered on Tm (bi-dentate attachment), ascertain that measured dynamics reflects the dynamics of protein backbone surface. Second, site-directed spin labeling allows to probe the dynamics of Tm at selected sites (in this work: N-terminus, mid-region, Tn binding region, and C-terminus). Saturation transfer electron paramagnetic resonance (ST-EPR) was the method of choice to probe the dynamics of Tm on actin. There was a gradient of mobility of Tm along its length in the muscle fiber with the C-terminus position being less mobile than the other three positions. Presence of Tn decreased the dynamics of Tm with no significant effect of Ca^2+^ and S1.

## Materials and Methods

### Protein Preparation

#### A. Tropomyosin

WT skeletal α-Tm plasmid was a generous gift from Dr Louise Brown (Macquarie University, Australia). L13C/N17C, H153C/D157C, G188C/E192C and K268C/E272C (referred to as 13/17, 153/157, 188/192 and 268/272 respectively in the paper) were engineered with the Stratagene QuickChange® II kit protocol (Stratagene, Agilent Technologies, Santa Clara, CA), the fidelity of the mutagenesis was confirmed by DNA sequencing. The pJC20 plasmid (American Type Culture Collection, Manassas, VA) containing the Tm was transformed into *E. coli* BL21 (DE3) cells (QIAGEN, Valencia, CA) and grown at 37°C in Luria-Bertani (LB) broth (USB Corporation, Affymetrix, Santa Clara, CA) containing 50 µg/mL ampicilin (Fisher Scientific, Pittsburg, PA) to an optical density of 0.6 at 595 nm. Protein expression was induced by the addition of 1 mM isopropyl β-D-thiogalactoside (IPTG) (Anatrace Incorporation, Maumee, OH) and cells were grown for 4 to 5 hours. The cells were harvested by centrifugation at 6,000 g for 15 minutes and re-suspended in 150 mM NaCl, 5 mM EDTA, 20 mM Tris-HCl, pH 7.5. The re-suspended pellet was sonicated (two pulses of 80 Watts each for 1 min each time with 1 min interval) and heated for 10 min at 80°C. After cooling on ice for an hour, the cell debris was removed by centrifugation at 15,000 g for 15 min. The supernatant's pH was lowered to 4.5 to precipitate the Tm and centrifuged at 15,000 g for 15 min. The pellet was re-suspended in Tm buffer A (100 mM NaCl, 5 mM sodium phosphate, pH 7.0), and the pH is adjusted to 7.0 with NaOH. Tm was loaded on a 20 mL Q Sepharose™ Fast Flow Column (Amersham Biosciences, GE Healthcare Life Sciences, Piscataway, NJ) and eluted using a salt gradient of 6 mM/min from 0.1 M NaCl (10% Tm buffer B (5 mM sodium phosphate, 1 M NaCl, pH 7.0) to 0.4 M NaCl (40% Tm buffer B) at a flow rate of 1.5 ml/min. Tm eluted between 0.25 M and 0.35 M NaCl depending on the mutant. The purified Tm mutants were treated with 10 mM DTT on ice for 1 hour. DTT was removed from solution by 12 hrs dialysis against three changes of 300 mL degassed Tm buffer A, at 4°C. Following dialysis Tm was incubated with 3-fold molar excess of 3,4-Bis-(methanethiosulfonylmethyl)-2,2,5,5-tetramethyl-2,5-dihydro-1H-pyrrol-1-yloxy spin label (referred to as HO-1944 in the paper) [18], [19] for 16 hrs at 4°C. Labeled Tm was dialyzed against three changes of 300 mL Tm buffer A to remove excess label. The efficiency of labeling was estimated by double integration of EPR signal. Only samples that were more than 80% labeled were used in subsequent experiments.

#### B. Troponin complex

Crude skeletal Tn (skTn) complex was extracted from rabbit skeletal muscle acetone powder (Pel-Freeze Biologicals, Rogers, AR) overnight with a 15∶1 (v/w) ratio of 1 M KCl, 25 mM Tris pH 8.0, 0.1 mM CaCl_2_, and 0.1 mM DTT. The extract was centrifuged at 11,000 g for 10 min at 4°C. The pellet was purified as described in [20] to separate pure skTn from Tm and other associated proteins.

#### C. Actin

Actin was extracted from rabbit skeletal muscle acetone powder (Pel-Freeze Biologicals) at 0°C for 30 min with G-actin buffer (2 mM Tris-HCl pH 8.0, 0.2 mM ATP, 0.5 mM β-mercaptoethanol, and 0.2 mM CaCl_2_). The extract was centrifuged at 10,000 g for 1 hour to separate the extracted actin from myosin. 50 mM KCl, 2 mM MgCl_2_ and 1 mM ATP were added to polymerize actin. Solid KCl was slowly added with gentle stirring to the polymerized actin solution to a final concentration of 0.6 M. The mixture was left on ice for 1.5 hours and then centrifuged at 80,000 g for 3 hours to isolate actin. G-actin was obtained after re-suspension of the pellet with G-actin buffer (3 ml of G-actin buffer per gram of acetone powder used), overnight dialysis against G-actin buffer and clarification by centrifugation at 50,000 g for 3 hours. G-actin was polymerized with addition of solid KCL to a final concentration of 50 mM [21]. A typical actin preparation yielded 15 mg of actin per gram of muscle acetone powder.

#### D. Myosin, S1 and HMM

Myosin was extracted from New Zealand rabbit back and leg muscle in high salt buffer(0.5 M KCl, 10 mM potassium phosphate, pH 6.5) as described in [22]. Heavy Meromyosin (HMM) was prepared by tryptic digestion of myosin. Trypsin (Sigma-Aldrich, St. Louis, MO) was added to a final concentration of 0.05 mg/mL to a 2% solution of myosin for 5 min. The digestion was stopped by adding 0.1 mg/mL of trypsin inhibitor PMSF (Sigma-Aldrich) and dialyzed overnight against 0.02 M KCl, 10 mM potassium phosphate, pH 6.5, and 1 mM DTT. HMM was separated from LMM by centrifugation at 50,000 g for 1 hour [23].

Subfragment-1 (S1) of myosin was extracted by chymotryptic digestion of myosin. α-chymotrypsin (Sigma-Aldrich) was added to a final concentration of 0.05 mg/mL to a 2% solution of myosin for 10 min. The digestion was stopped by adding 0.1–0.3 mM of PMSF [24].

#### E. Muscle fiber

Psoas muscle fibers were extracted from New Zealand rabbit in a cold room with the carcass on ice. The psoas muscle was first stripped from its outer membrane using fine forceps and the fibers separated into 3 mm diameter bundles. The bundles were tied to a stick and washed with rigor buffer (60 mM KAc, 25 mM MOPS, 2 mM MgCl_2_, 1 mM EGTA, 1 mM NaN_3_, pH 7.0) (referred to as RB). The fibers were shaken for 24 hours in a cold room in fiber glycerination buffer (75% RB, 24.5% glycerol, 0.5% Triton X-100, 9 mM EGTA). Final glycerination was accomplished by 24 hours incubation in fiber storage buffer (50% RB, 50% glycerol) followed by storage at −20°C until further use (for 3 to 6 months).

Once needed, 3–5 single fibers were dissected from the stored fiber bundles. Sarcomeric proteins, myosin, Tn, and Tm were removed from the fibers by washing with Hasselbach-Schneider solution (0.8 M KCl, 4 mM EGTA, 20 mM sodium phosphate, 10 mM MgATP, pH 6.4) for 6 hours with shaking at 4°C (the depleted fibers will be referred to as “ghost” fibers) (Figure S1, lane 2). Labeled Tm was added at concentrations of 20 µM to the “ghost” fibers in RB buffer for 15 hours at 4°C (Figure S1, lane 3), followed by reconstitution with 20 µM troponin complex and 20 µM S1 when needed (Figure S1, lane 4). Unbound proteins were removed by repeated washes with rigor buffer. The efficiency of reconstitution was verified by 12% SDS gel electrophoresis.

The stochiometry of reconstituted fibers was estimated by quantification of the SDS-PAGE gels (Figure S1). The efficiency of labeled Tm reconstitution in the ghost muscle fiber is inferred from lane 3. The ratio of labeled Tm∶actin in the reconstituted muscle fiber is 1∶4.5 while that of Tm∶actin in the native fiber is 1∶3. The efficiency of Tn reconstitution was estimated from the TnI band (the only well resolved troponin subunit on the gel). The ratio of TnI∶actin in the reconstituted muscle fiber is 1∶4.5 compared to 1∶3 intrinsic actin∶TnI in native fiber. Finally, the ratio of the S1∶actin intensity in reconstituted ghost fibers is 1∶1, comparable to the ratio of intrinsic myosin∶actin in native fibers. Taking the efficiency of staining in consideration, the amount of S1 in the ghost fiber is therefore equal to twice the amount of myosin heads in the intrinsic fiber ∼480 uM.

The co-localization of Tm and actin in the reconstituted muscle fiber was verified by fluorescence confocal microscopy of EAM-labeled Tm146 (Figure S2). Fluorescently-labeled Tm was seen forming striations of 2 µm in length bisected by an unstained line. Separation of those lines was 2.5 µm identifying them as Z-lines. The 2 µm length of the Tm bands corresponds exactly to the length and position of actin filaments, thus the reconstituted Tm is associated only with actin in the ghost fibers.

This study was carried out in strict accordance with the recommendations in the Guide for the Care and Use of Laboratory Animals of the National Institutes of Health. The protocol was approved by the Animal Care and Use Committee (ACUC) at Florida State University (FSU). Protocol #: 9134 and 1105. All surgery was performed under sodium pentobarbital anesthesia, and all efforts were made to minimize suffering.

### Circular Dichroism

#### A. Wavelength scan

Bi-functionally labeled Tm were dialyzed into 60 mM NaF, 20 mM sodium phosphate, pH 7.0 and diluted to 0.2 mg/mL. NaF was used instead of NaCl to minimize contribution to the CD spectra. Measurements were performed on an AVIV- 202 CD spectrometer (AVIV Biomedical, Lakewood, NJ). The samples were measured in a 0.1 cm path length quartz cuvette (10 mm×10 mm), using a temperature regulated cuvette holder, in a N_2_ atmosphere. CD scans between 195 nm and 160 nm were performed in triplicate in steps of 0.3 nm at the rate of 1 nm per second. Ellipticity (θ) values were converted to mean residue ellipticity (θ_(MRE)_):
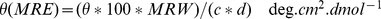
with MRW the mean residue weight, c the concentration in mg/mL, and d the optical path length in cm.

#### B. Melting curves and enthalpy

Thermal denaturation was followed by CD at 221 nm (diagnostic wavelength for α-helices). The denaturation curves were obtained by varying the temperature from 15°C to 90°C in steps of 5°C, adequate for transitions with 20°C width. Melting temperature (T_m_) and van't Hoff enthalpy (ΔH_m_) were calculated from the denaturation curves as described in [25], [26].

### *In-vitro* Motility

An *in-vitro* motility assay measures the rate of movement of fluorescently labeled F-actin sliding on heavy meromyosin (HMM). The functionality of labeled Tm mutants was assayed by infusion of 10 nM Tm, 10 nM Tn and 8 nM of labeled actin into a flow cell containing the HMM deposited at 250 µg/mL on a nitrocellulose-coated cover-slip. The assays were performed in 2 mM ATP, 16.7 mM glucose, 100 µg/ml glucose oxidase, 18 µg/ml catalase and 40 mM DTT. Excess unbound protein is washed with 25 mM imidazole, 25 mM KCL, 4 mM MgCl_2_, 1 mM EGTA, pH 7.0. The movement of rhodamine-phalloidin labeled actin at 30°C was recorded in 45 seconds clips using a VE1000SIT camera (Dage-MTI, Michigan City, IN) and analyzed using IVM program (Dr. Michael Regnier, University of Washington). The assay was done in collaboration with Dr. P.B. Chase's laboratory (Florida State University).

### Mass Spectrometry

The efficiency and mode of bi-functional label modifications was determined by Fourier Transform Ion Cyclotron Resonance (FT-ICR) mass spectrometry. The purified mutants at 10 µM were desalted using C4 ZipTip® (Millipore, Billerica, MA) with 50∶50∶1 water: acetonitrile: formic acid and injected into a hybrid 14.5 T LTQ-FT-ICR mass spectrometer by direct infusion micro-electrospray ionization using a syringe pump with a constant flow (0.5 µL/min). 200 time-domain transients were collected for each sample. The measurements and analysis were performed by George Bou-Assaf in Dr. Allan Marshall's laboratory (National High Magnetic Field Laboratory – Florida State University).

### Electron Paramagnetic Resonance

#### A. Continuous wave EPR (cw-EPR)

Cw-EPR experiments were performed on a Bruker ECS-106 spectrometer (Bruker Biospin Corporation, Billerica, MA) in a TE_102_ cavity at room temperature (22°C). The cw spectra were recorded at a microwave power of 8 mW and modulation amplitude of 1 G. The analysis of the spectra was performed using Labview EPR developed in our laboratory.

#### B. Saturation transfer EPR (ST-EPR)

In ST-EPR experiments, the 90° out-of-phase second harmonic spectra (V'_2_) was recorded at a microwave field of 0.25 G and modulation amplitude (H_m_) of 5 G as calculated from cavity Q with 1 mg/mL of peroxylamine disulfonate as described in [27]. The temperature was controlled at 4°C by flowing pre-cooled N_2_ gas through the radiation slits into the cavity. The experiments were performed in 60 mM KAc, 25 mM MOPS, 2 mM MgCl_2_, 1 mM EGTA, 1 mM NaN_3_, pH 7.0.

The V'_2_ spectra were integrated and normalized to the spin label concentration as determined from in-phase spectra collected under non-saturating conditions, microwave field of 0.144 G and H_m_ of 1 G. The normalized integral of V'_2_ was compared to the calibration curve obtained from spectra of spin labeled hemoglobin (Hb) in 87.55% weight/weight of Hb-glycerol mixture. Hb is undergoing Brownian diffusion with a rotational correlation time (τ_r_) given by the Stokes-Einstein equation (τ_r_ = πηr/k_B_T) where k_B_ is the Boltzmann's constant, T the temperature, η the viscosity and r the radius of the protein. A general description of the experimental procedures and calibration can be found in [27], [28].

## Results

### Bi-functional labeling of Tm

**Figure 1:**
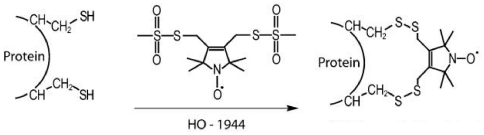
Spin labeling of a protein with the bi-functional spin label HO-1944. HO-1944 attaches itself via two disulfide bonds to two cysteines separated by 4 residues.

To probe the dynamics of Tm backbone directly, without the overlaying motion of a spin label we have employed a bi-functional spin label HO-1944 (Figure 1) that binds to two cysteines in double cysteine (i,i+4) mutants of Tm. The bi-dentate attachment of the spin label was verified by Fourier Transform Ion Cyclotron Resonance Mass Spectrometry. The labeled Tm mutants had two populations of masses separated by 230±3 Da, 231±3 Da, 228±3 Da, 229±3 Da for 13/17, 153/157, 188/192, 268/272 respectively. The expected separation between bi-dentate labeled Tm and non-labeled Tm is of 228 Da. No population corresponding to mono-dentate labeled Tm (308 Da mass increase) was observed.

### Structure, function and spectroscopic characterization of the bi-functionally labeled Tm

#### A. Structural characterization of the bi-functionally labeled Tm

**Figure 2:**
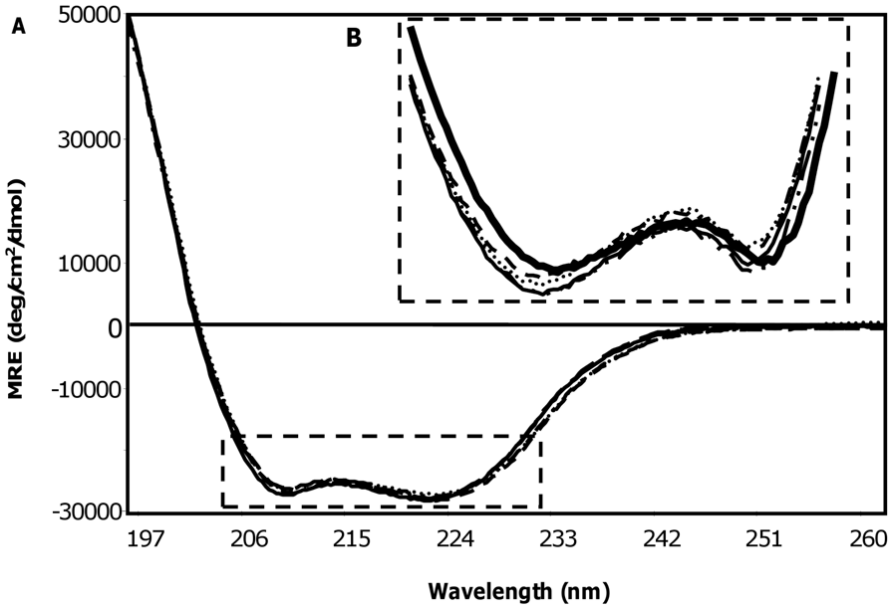
Circular Dichroism of the labeled Tm. **A.** Mean residue ellipticity of the labeled Tm mutants, 13/17 (*thin solid line*), 153/157 (*dashed line*), 188/192 (*dotted line*), 268/272 (**- ^.^^.^ -**) and WT Tm (*thick solid line*). **B.** Magnified area of **A.**

The secondary structure of the bi-functionally labeled Tm was assessed using circular dichroism (CD). CD spectra of the four bi-functionally labeled Tm positions (13/17, 153/157, 188/192, and 268/272) were compared to that of the WT Tm spectrum (Figure 2). There is less than 3% change in mean residue ellipticity (MRE) at 195 nm and 208 nm (the two characteristic wavelengths for α-helices) for any of the four Tm mutants (Figure 2). Thus, mutagenesis and labeling did not result in global unfolding of tropomyosin.

**Figure 3:**
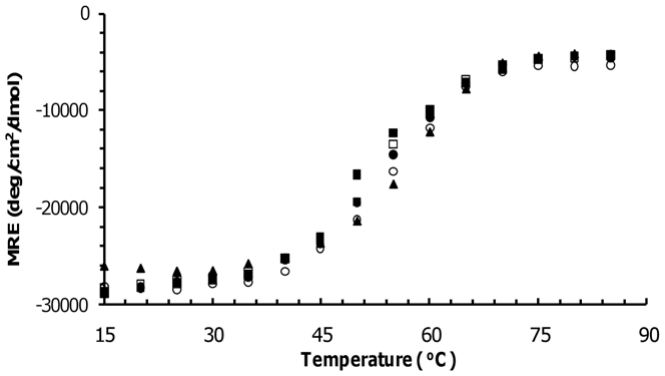
Thermal denaturation of labeled Tm. Mean residue ellipticity of Tm 13/17 (*filled square*), 153/157 (*open square*), 188/192 (*open circle*), 268/272 (*filled circle*), and WT Tm (*filled triangle*) at 221 nm as a function of temperature.

The possibility of the observed 3% difference affecting thermodynamic stability was assessed by thermal denaturation as shown in Figure 3. The melting curves of the bi-functionally labeled Tm exhibited a small leftward shift in the melting temperature between 2±4°C and 6±4°C as compared to the WT Tm indicating some loss in stability (Figure 3). Our dynamics measurements are performed 40°C below the unfolding temperature. Thus, the small effects in melting temperature are unlikely to translate into significant dynamics changes at this temperature.

The change in enthalpy (ΔH_m_) was obtained from the slope of the ratio of denatured over non-denatured protein (−R ln(f_denat_/f_non-denat_)) as function of temperature (1/T) [25]. ΔH_m_ of the bi-functionally labeled Tm 13/17, 153/157, 188/192 and 268/272 differed from ΔH_m_ of WT Tm by 5±5, 6±5, 6±5 and 2±5 kcal/mol respectively (Table 1). These differences in ΔH_m_ are comparable to single cysteine substitutions of solvent-exposed sites of the α-helix of T4-lysozyme [29].

**Table 1:** Melting temperature and enthalpy of the labeled Tm.

Sample	Melting Temperature (°C)	Enthalpy (kcal/mol)
WT Tm	57±2	52±5
13/17*	51±2	47±5
153/157*	53±2	46±5
188/192*	55±2	58±5
268/272*	54±2	54±5

*Labeled with HO-1944.

#### B. Functional characterization of the bi-functionally labeled Tm

**Figure 4:**
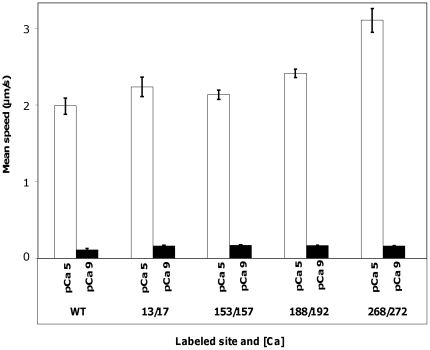
*In-vitro* motility of the labeled Tm. Mean speed of regulated thin filaments reconstituted with WT Tm and the four labeled Tm: 13/17, 153/157, 188/192 and 268/272 under high calcium concentration, pCa 5 (*open bar*) and low calcium concentration, pCa 9 (*filled bar*).

To assess potential deleterious effects of mutagenesis and labeling on function of Tm we have determined the extent of motility regulation by the bi-functionally labeled Tm in the *in-vitro* motility assay. As shown in Figure 4, there was no significant change in the mean speed of the actin filaments when any of the four bi-functionally labeled Tm was introduced into the thin filament as compared to the WT control under low calcium (pCa 9) and high calcium (pCa 5) condition. At pCa 5 the mean speed of actin movement in a regulated *in-vitro* motility assay was 2.2–3.1 µm/s for the labeled mutants as compared to 2±0.1 µm/s for WT Tm (Figure 4) and to 1.8±0.1 µm/s in previous studies [30]. For the inhibited state, at low Ca^2+^ concentration (pCa 9), the mean speed of the actin filaments was 0.16 µm/s for mutants as compared to 0.12±0.01 µm/s for WT (Figure 4) and to 0.1±0.01 µm/s in previous studies [30]. The fraction of filament moving at pCa 5 varied between 0.7 and 0.9 for the four Tm mutants studied and was comparable to the fraction of filaments moving for WT Tm (0.7).

#### C. Spectroscopic characterization of the labeled Tm

The prerequisite to measurements of protein dynamics using external probes is to restrict probe motion - in most extrinsic probes the signal originates more than 5 single bonds away from the backbone of the protein. To make sure that the measured motion is not that of the spin label with respect to the surface of a protein, the labeled Tm was cross-linked to DITC glass beads. The protein cross-linked to beads is not expected to exhibit any large scale motion (timescale less than milliseconds); any remnant motion will be that of spin label with respect to protein. The cross-linked Tm was monitored for librational motion of the spin label in the fast 1 ps–100 ns (by cw-EPR) and slow 1 µs–1 ms (by ST-EPR) motional regimes.

**Figure 5:**
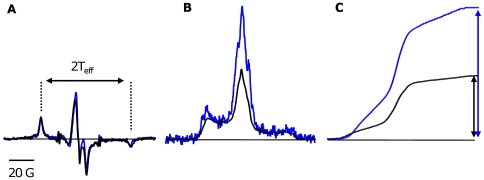
Dynamics of the labeled Tm in the muscle fiber. **A.** Overlay of the cw-EPR spectra of labeled Tm in ghost fiber *(black*) and labeled Tm cross-linked to DITC glass beads in solution (*blue*). The double arrows correspond to the hyperfine splitting (2 T_eff_) of the cw spectra. **B.** Overlay of the ST-EPR spectra of labeled Tm in ghost fiber (*black*) and labeled Tm cross-linked to DITC glass beads in solution (*blue*). **C.** Comparison of the normalized first integral of the ST-EPR spectrum (V'_2_) of labeled Tm in ghost fiber (*black*) and labeled Tm cross-linked to DITC-glass beads (*blue*). The double arrows correspond to the height of the first integral of the normalized V'_2_ spectra.

The cw-EPR spectra of the bi-functionally labeled Tm cross-linked to DITC glass beads have a splitting (2 T_eff_) of 70 G (Figure 5A), equal to the rigid-limit values [31]. The ST-EPR spectra of the bi-functionally labeled Tm cross-linked to DITC glass beads have an effective correlation time in the microsecond timescale, again equal to the rigid-limit (Figure 5B and Figure 5C) [32]. Therefore there is no librational motion of the spin label with respect to the surface of the protein for both cw-EPR and ST-EPR. When introduced into the ghost fiber, the nanosecond dynamics is completely inhibited, the splitting of cw-EPR spectra is 70 G, same value as a rigid-limit spectrum of Tm cross-linked to DITC glass beads (Figure 5A). Therefore, the motion of Tm in the muscle fiber is outside cw-EPR sensitivity and thus should be monitored by ST-EPR which is suitable for slower rotational correlation times. The ST-EPR spectrum of labeled Tm in ghost fiber is within ST-EPR sensitivity as seen compared to Tm cross-linked to DITC glass-beads (Figure 5B and Figure 5C).

**Figure 6:**
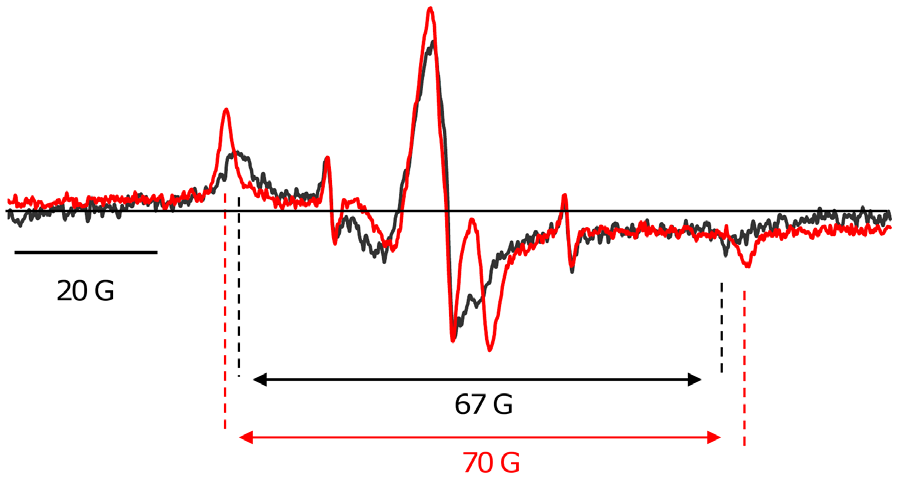
Dynamics of the labeled Tm in solution and in the muscle fiber. Overlay of the cw-EPR spectrum of Tm in solution (*black*) versus Tm in the muscle fiber (*red*). The double arrow defines hyperfine splitting of the cw spectra.

In solution, isolated Tm cw-EPR spectrum has a hyperfine splitting of 67±1 G (Figure 6). This hyperfine splitting corresponds to an effective correlation time of 40 ns as calculated from: τ_r_ = a (1–2 T_eff_/2 T^R^_eff_)^b^ with 2 T_eff_ and 2 T^R^_eff_ denoting respectively the observed and the rigid limit hyperfine splitting values [33].

ST-EPR was employed to probe slower, microsecond dynamics. The reconstituted Tm exhibits a smaller intensity when compared to Tm cross-linked to DITC beads, qualitatively showing evidence for sub-millisecond motions (Figure 5B and Figure 5C).

### Differential dynamics along Tm length in the muscle fiber

ST-EPR was performed of the four labeled Tm positions reconstituted in the muscle fiber under the various conditions studied (with/without Tn, with/without Ca^2+^, with/without S1) (Figure S3).

**Figure 7:**
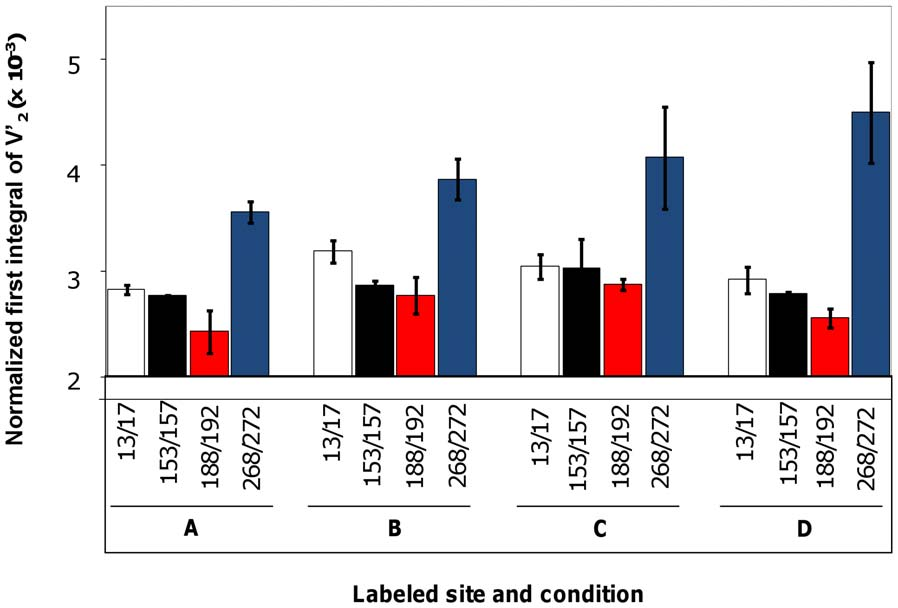
Gradient of Tm dynamics along its length. Normalized first integral of V'_2_ spectra of 13/17 (*white bar*), 153/157 (*black bar*), 188/192 (*red bar*), and 268/272 (*blue bar*) in ghost fiber (**A**), in presence of Tn (**B**), in presence of Ca^2+^(**C**), and decorated with S1 (**D**).

The effective correlation time of the protein is obtained by comparing the normalized first integral ST-EPR spectrum to the calibration curve of spin labeled hemoglobin (Hb). The values of the normalized integral of ST-EPR for each of the mutants in the absence and presence of Tn, Ca^2+^ or S1 are shown in Figure 7.

In the partially reconstituted muscle fiber (i.e. only reconstituted with the labeled Tm), 268/272 has an effective correlation of 830 µs±40 µs which is six to fifteen times slower than the other three mutants (130 µs±20 µs, 120 µs±10 µs, and 60 µs±30 µs for 13/17, 153/157, and 188/192 respectively) (Table 2). This gradient of mobility persisted in the presence of Tn, Ca^2+^ and S1. Thus, the C-terminus of Tm appears to have a longer effective correlation time compared to the other three regions in any condition studied, and thus is less flexible.

**Table 2:** Effective correlation time of the labeled Tm.

Position & Condition	Correlation Time (**µ**s)	Error (**µ**s)
**13/17**	130	20
+ Tn − Ca^2+^	340	90
+ Tn + Ca^2+^	240	70
+ Tn + Ca^2+^ + S1	170	10
**153/157**	120	10
+ Tn − Ca^2+^	150	20
+ Tn + Ca^2+^	270	160
+ Tn + Ca^2+^ + S1	120	10
**188/192**	60	30
+ Tn − Ca^2+^	130	50
+ Tn + Ca^2+^	150	20
+ Tn + Ca^2+^ + S1	70	10
**268/272**	830	40
+ Tn − Ca^2+^	2000	900
+ Tn + Ca^2+^	3000	2100
+ Tn + Ca^2+^ + S1	5000	3000

### Effect of troponin, Ca^2+^, and myosin subfragment-1 on the dynamics of Tm in the muscle fiber

The presence of troponin in the muscle fiber exhibited a 3-fold increase in effective correlation time of the 13/17 position. A 1.5 to 2-fold increase was observed in the two mid-region positions 153/157 and 188/192 and in the C-terminus position 268/272 (Table 2). Such an effect is consistent with Tn clamping down on Tm and increasing its interaction with actin filament. Surprisingly, Ca^2+^ had no effect of dynamics of Tm in any of the regions probed (Table 2). We hoped to see changes in Tm dynamics induced on filament activation but the effective correlation time does not vary by more than 30% on average in the presence or absence of Ca^2+^ for any of the probed sites. Finally, in the presence of saturating S1 concentration, there was little if any effect (the movement was slightly faster ∼33%) but within the errors of our measurement) (Table 2).

## Discussion

The present study takes advantage of bi-dentate attachment of bi-functional labels to investigate the dynamics of Tm in the N-terminus, troponin binding region, and C-terminus. For all four sites we find that Tm in solution moves rapidly with an effective correlation time of ∼40 ns, but when introduced in the “ghost” muscle fibers the motion decreases by three orders of magnitude. In muscle fibers Tm exhibits motional gradient with the C-terminus of Tm less dynamic than other regions (5∼15-fold). Presence of troponin in muscle fibers further decreases mobility (1.5∼3-fold) of all four Tm sites. Unexpectedly, Ca^2+^ and S1 had no significant effect on Tm dynamics.

Tm plays an essential role in the regulation of muscle contraction. With the other main thin filament protein, Tn, Tm is responsible for the cooperative calcium-dependent regulation of actin-myosin interaction. The regulatory system has been shown to have three states that can be biochemically differentiated known as *blocked*, *closed* and *open* [4]. These states appear to be related to the three different structural states of Tm position on the thin filament observed in EM reconstructions [7]. The positioning of Tm on the actin filament and thus the degree of exposure of the myosin-binding site on actin is dependent on the flexibility of the Tm. One approach to evaluate the flexibility of a molecule is by measuring its persistence length. Tm was shown to be a semi-flexible coiled-coil with a persistence length of 130±40 nm [34], [35], [36], [37]. Since the length of tropomyosin is ∼40 nm, this means that “information” perceived by one tropomyosin is passed to the neighboring tropomyosin. This relay of “information” (in this case the position of Tm on F-actin) is the basis of the cooperative effect of Tm in the thin filament. Recently, a molecular dynamics simulation showed that Tm has a persistence length four times longer than (∼430 nm) what was originally thought [38]. Tm is pictured in this case as a semi-rigid coiled-coil. Either way, being a semi-flexible or semi-rigid coiled coil, the fluctuations of Tm (i.e. change in Tm dynamics) might lead to a change of Tm position on actin exposing or hiding myosin-binding sites on actin. Indeed, a model involving the dynamics of Tm has been proposed, in which variations in the amplitude of tropomyosin vibrations (its dynamics) drive the change in its radial position on actin [14].

Numerous studies characterized various aspects of tropomyosin dynamics in solution. A fluorescent study showed that Tm is flexible in solution (ns timescale) [37]. We observed a similar timescale of motion for isolated Tm (40 ns). Flexibility of Tm can be evaluated from its persistence length, a measure of lengthwise thermal bending fluctuations. Capillary viscosity measurements showed that Tm is semi-flexible with a persistence length of 1300 Å±40 Å [34]. Birefringence showed that the protein could be equally well modeled as a rigid rod or as a semi-flexible rod with a persistence length of 1500 Å [36]. Crystal structures of Tm showed evidence of disorder and possibility of motion [35], [39], [40]. An analysis of x-ray diffuse scatter in Tm crystals at different temperatures was attributed 8 Å fluctuations of C-terminus [41], [42].

More direct measurement of protein dynamics by NMR showed differential flexibility of Tm along its length with the C-terminus overlap region being unstructured and flexible [43], [44]. Molecular dynamics (MD) simulations gave further support to the idea of differential flexibility with C-terminus moving twice as fast as the N-terminal region [45]. Thus, there is consensus of motional gradient in tropomyosin with the C-terminus of Tm being more dynamic that the rest; however all the above studies were performed for isolated Tm i.e. not associated with actin. As observed here, Tm interacting with actin in the reconstituted fibers exhibit different dynamics than in solution (thousands-folds decrease). There is still a gradient of mobility with the C-terminus of Tm being least mobile [43], [44]. The decrease in dynamics of the C-terminus region is not due to a local effect i.e. it is not due to the immobilization of the spin label by the proximity to actin surface. The four positions to which the spin label is attached are oriented in the same fashion with respect to actin [46]. Additionally, because the label is placed on each of the two strands of Tm, at every position and in every state, one cysteine is pointing towards the actin and the other cysteine on the other strand is pointing away from F-actin (Figure S4). Therefore, if there is any local effect of spin label immobilization on the actin surface, we should observe two populations: one slow (the strand having the spin label pointing towards actin) and one fast (the strand having the spin label pointing away from actin). We do not observe two populations of dynamics for any position and in any state studied indicating no local immobilization of spin label by actin. Labels at positions 153/157, 188/192, 268/272 should experience the same degree of steric restriction (the N-terminal site 13 is still facing actin surface but is at longer distance from the surface). Therefore, the differences in mobility cannot be attributed to the differential interactions of the four Tm positions with actin. Thus, the EPR spectra are not reporting on the steric restraints imposed on side chains and the variation of dynamics along the length of Tm is a property of backbone itself.

Less is known about the dynamics of Tm in the thin filament. The original attempt to measure Tm rates by time resolved fluorescence anisotropy was hampered by the fluorescent lifetimes that were 10–50 times shorter than the correlation times they were supposed to measure [37]. EPR [16] and phosphorescence [17] studies extended the time-window of the methods to match the protein dynamics but the quantification of the backbone dynamics was difficult since the probes were attached at a single site and the signals were composites of the label motion and backbone motion, the observed dynamics of Tm was orders of magnitude faster (40 ns [16], 2 µs [17], [37] than the motion of F-actin (70 µs [31], [47], [48], [49]). Naturally, the Tm can move independently of actin but with bi-functional label used here Tm mobility is similar to that of actin suggesting strongly that singly attached probes used before were sensitive to local, librational motion.

Once in the thin filament Tm dynamics can be affected by other components and ligands: Tn, Ca^2+^ and S1. Tn binding resulted in a decrease in Tm dynamics at all sites studied (N-term, mid-region, Tn binding site and C-term) of Tn binding. The mid-region is the site of troponin binding to Tm, as shown by FRET [50] and visualized in Tm-Tn co-crystals [51] which would explain the decrease in Tm dynamics at positions 153/157 and 188/192. The N-terminus of TnT is known to connect the binding region to the termini of Tm [50], [51], which would explain the decrease in Tm dynamics at positions 13/17 and 268/272.

Ca^2+^ binding, which regulates thin filament activation resulted in no significant change of Tm mobility. Addition of S1 that further activates the filament also did not induce changes in mobility. The latter result is somewhat different from that reported by phosphorescence anisotropy (two-fold increase of mobility) [17]. However, that observation was made in the absence of Tn while our motivation was to look for activating effects of S1 in a fully reconstituted system.

In conclusion, Tm has a thousands-fold decrease dynamics in the thin filament than when isolated due to its interactions with actin. The C-terminus position of Tm was observed to be less dynamic than the other three positions. Tn decreases Tm dynamics (anchoring effect) when present in the muscle fiber. Ca^2+^ and S1 have no significant effect on Tm dynamics. Thus, the change of Tm backbone dynamics is not a main factor in the movement of Tm on the actin filament from *blocked* to *closed* to *open* state and thus it is unlikely to play a role in the regulation of muscle contraction as proposed before [14].

## Supporting Information

Figure S1 **SDS-PAGE of muscle fibers.** *Lane 1*: native fiber; *lane 2*: “ghost” fiber; *lane 3*: “ghost” fiber reconstituted with labeled Tm; *lane 4*: reconstitution with Tn and addition of S1.
(TIF)

Figure S2 **Fluorescence confocal microscopy of tropomyosin labeled with EAM at position 146 reconstituted in “ghost” muscle fibers.** 63× magnification; z-line denoted by white arrows.
(TIF)

Figure S3 **ST-EPR spectra of the bi-functionally labeled Tm in the muscle fiber.** Overlay of the normalized first integral of V'_2_ (*thick line*) and the V'_2_ spectrum (*thin line*) of the four labeled Tm 13/17 (*first row*), 153/157 (*second row*), 188/192 (*third row*), and 268/272 (*fourth row*) in “ghost” fiber (*first column*), in presence of Tn (*second column*), in presence of Ca^2+^ (*third column*), decorated with S1 (*fourth column*).
(TIF)

Figure S4 **Geometry of the labeled Tm sites with respect to the actin filament.** Using a new actin-Tm model [46], the labeled Tm (A) site 13/17, (B) site 153/157, (C) site 188/192, and (D) site 268/272 are shown in blocked (*purple*), closed (*cyan*) and open (*yellow*) states. The position of the “I” site cysteine is shown in *green*.
(TIF)
