# Supplementary figures and images for: Dynamics of Tropomyosin in Muscle Fibers as Monitored by Saturation Transfer EPR of Bi-Functional Probe
Source: PLoS One. 2011 Jun 20;6(6):e21277. doi: 10.1371/journal.pone.0021277 (PMC3118794; doi:10.1371/journal.pone.0021277)

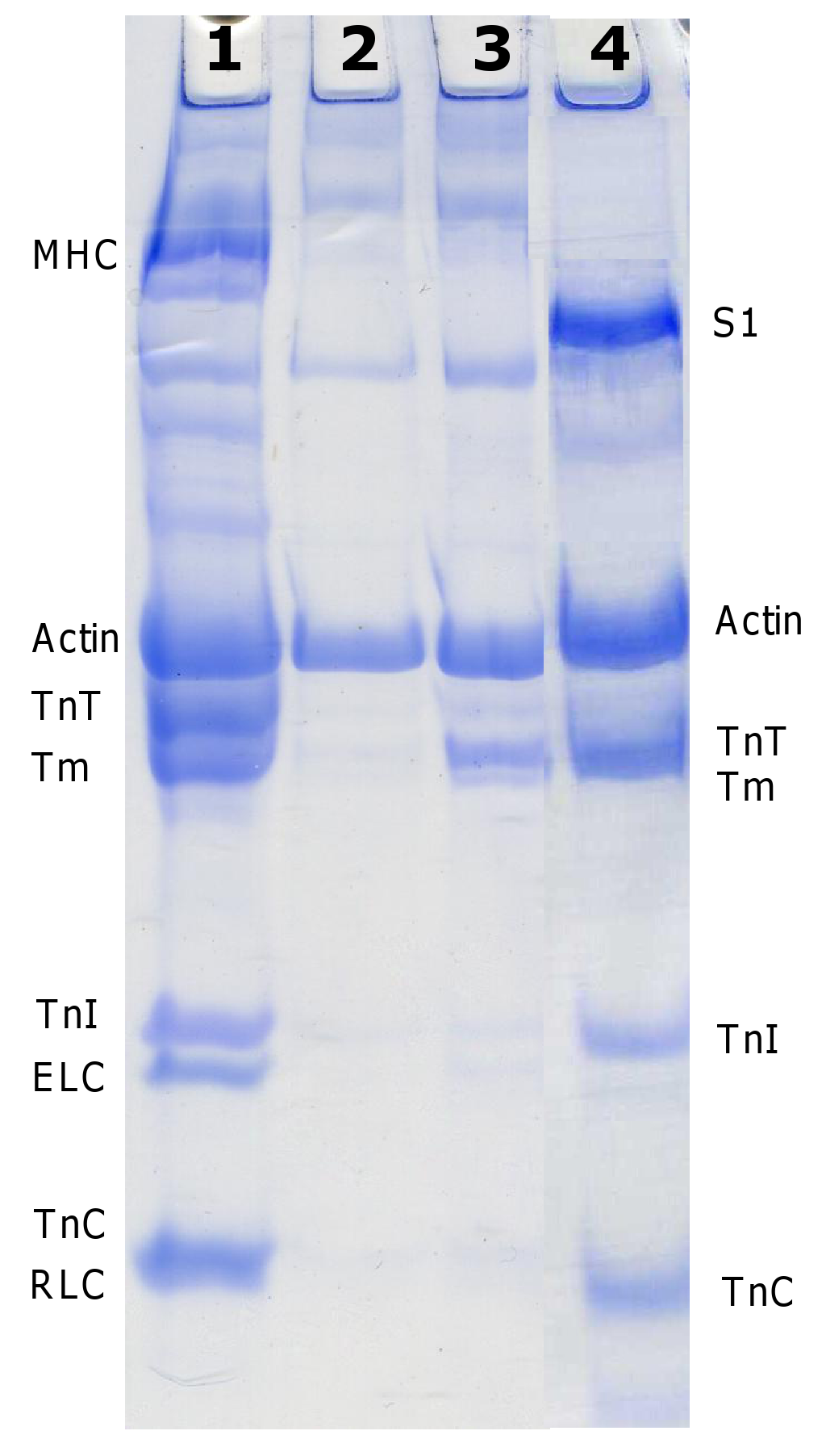

Supplement: Figure S1 — SDS-PAGE of muscle fibers. Lane 1: native fiber; lane 2: “ghost” fiber; lane 3: “ghost” fiber reconstituted with labeled Tm; lane 4: reconstitution with Tn and addition of S1. (TIF) [file pone.0021277.s001.tif]

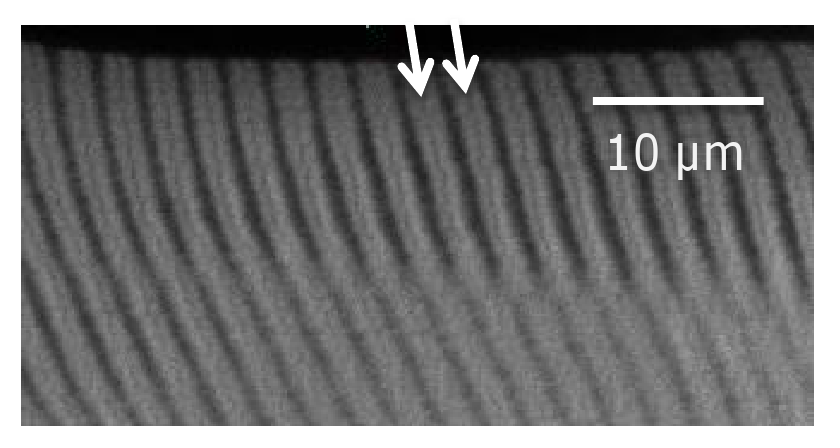

Supplement: Figure S2 — Fluorescence confocal microscopy of tropomyosin labeled with EAM at position 146 reconstituted in “ghost” muscle fibers. 63× magnification; z-line denoted by white arrows. (TIF) [file pone.0021277.s002.tif]

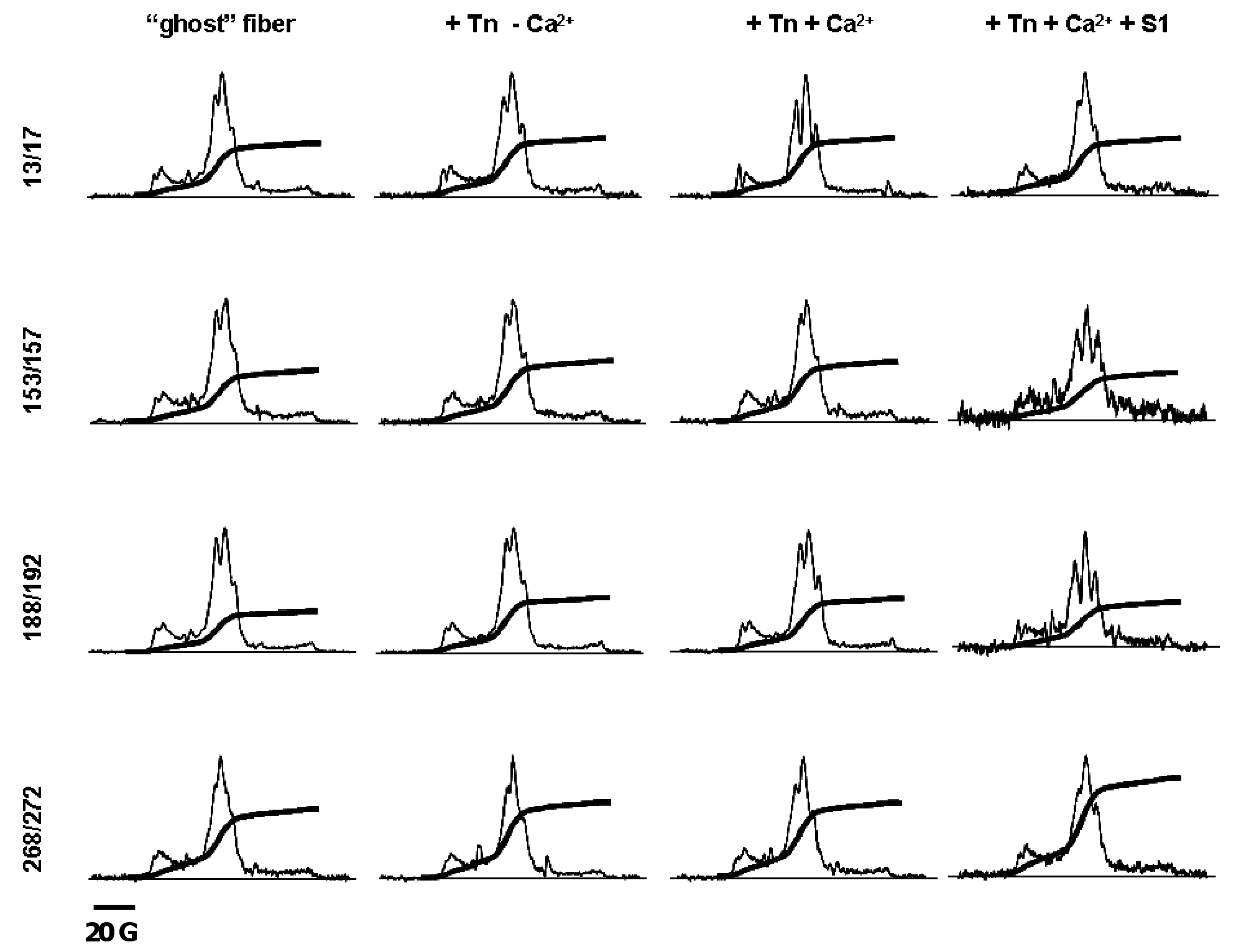

Supplement: Figure S3 — ST-EPR spectra of the bi-functionally labeled Tm in the muscle fiber. Overlay of the normalized first integral of V'2 (thick line) and the V'2 spectrum (thin line) of the four labeled Tm 13/17 (first row), 153/157 (second row), 188/192 (third row), and 268/272 (fourth row) in “ghost” fiber (first column), in presence of Tn (second column), in presence of Ca2+ (third column), decorated with S1 (fourth column). (TIF) [file pone.0021277.s003.tif]

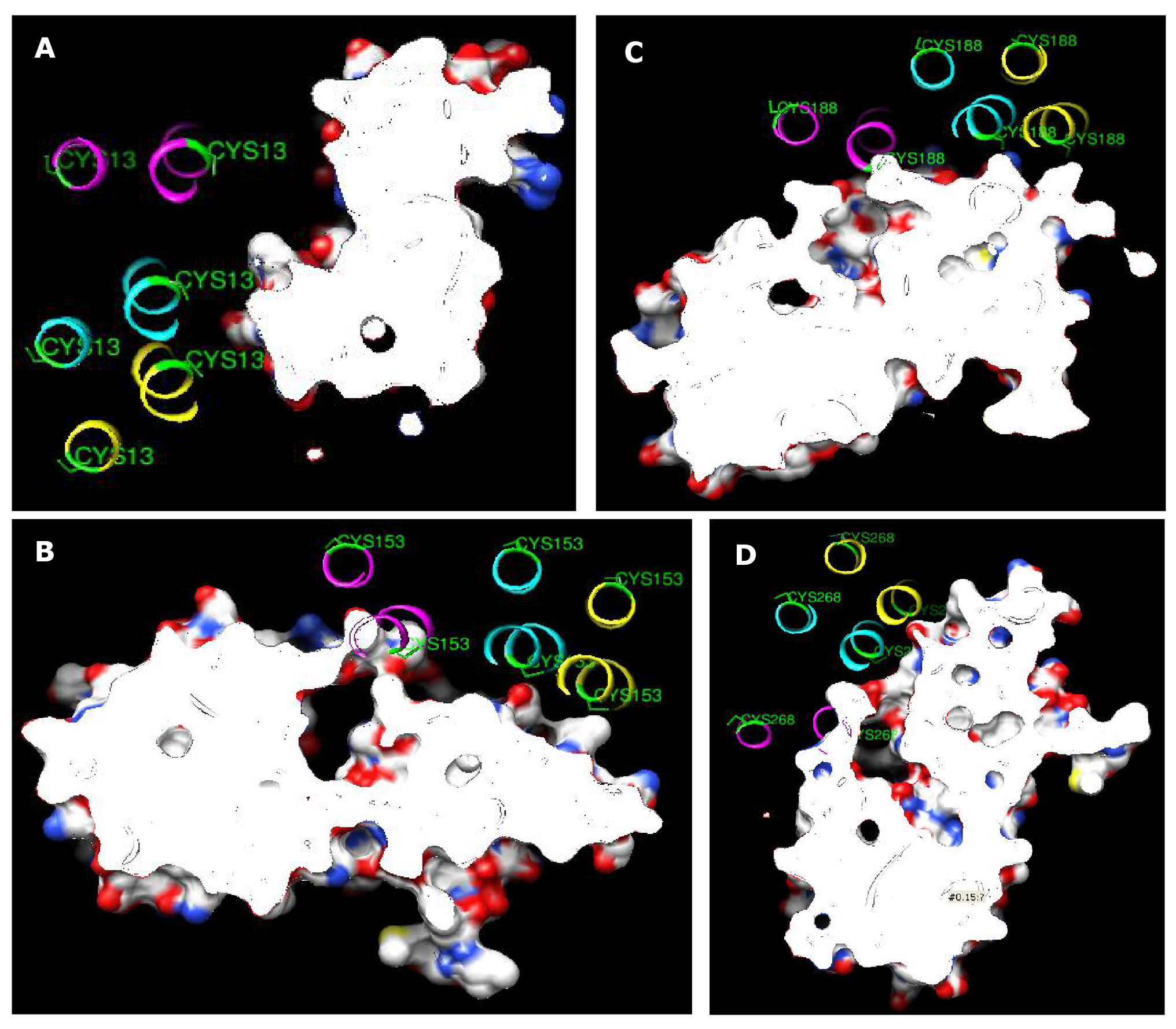

Supplement: Figure S4 — Geometry of the labeled Tm sites with respect to the actin filament. Using a new actin-Tm model [46], the labeled Tm (A) site 13/17, (B) site 153/157, (C) site 188/192, and (D) site 268/272 are shown in blocked (purple), closed (cyan) and open (yellow) states. The position of the “I” site cysteine is shown in green. (TIF) [file pone.0021277.s004.tif]
